# 亚实性肺结节CT阈值分割：实性成分识别与定量

**DOI:** 10.3779/j.issn.1009-3419.2017.05.07

**Published:** 2017-05-20

**Authors:** 文松 郑, 卿 王, 颖 王, 芳芳 郭, 欣悦 王, 铁链 于

**Affiliations:** 300052 天津，天津医科大学总医院医学影像科 Medical Imaging Department, Tianjin Medical University General Hospital, Tianjin 300052, China

**Keywords:** 亚实性结节, 腺癌, 计算机断层扫描, 阈值分割, 体积测量, Subsolid nodule, Adenocarcinoma, Computed tomography, Threshold segmentation, Volume measurement

## Abstract

**背景与目的:**

肺内亚实性结节（subsolid nodule, SSN）实性成分的识别与定量对SSN的鉴别诊断，病理预测和预后评估具有重要价值，但目前缺乏公认且客观的标准。本研究旨在应用计算机断层扫描（computed tomography, CT）阈值分割方法确定用于SSN实性成分识别与定量的最佳阈值。

**方法:**

回顾性分析102例SSN的CT图像。由观察者确定SSN内是否存在实性成分并借助软件确定实性成分的体积，以此作为参照标准。应用CT阈值分割方法对所有SSN进行分析，测量其内不同CT阈值对应的体素体积，假定该体积即为实性成分体积，计算实性成分与结节整体的体积比率。所得结果与相应参照标准进行比较，应用受试者工作特征曲线及*Wilcoxon*检验筛选最佳阈值。

**结果:**

阈值设为-250 HU时，识别SSN内实性成分的诊断价值最大（曲线下面积为0.982），以体积比率1.10%作为确认实性成分存在的界限值，其诊断效能最高。阈值设为-300 HU时诊断效能次之，对应曲线下面积和体积比率界限值分别为0.977、6.14%。阈值设为-250 HU、-300 HU时所得实性成分中位体积分别为202.7 mm^3^（598.2 mm^3^）、247.1 mm^3^（696.0 mm^3^），与参照标准199.5 mm^3^（743.1 mm^3^）间无显著差异（*P*=0.125, 1, 0.061, 3）。

**结论:**

CT阈值分割方法可对SSN实性成分进行准确识别与定量，阈值可设为-250 HU或-300 HU。

## Abstract (en)

**Background and objective:**

The detection and quantification of solid components in pulmonary subsolid nodules (SSN) are of vital importance on differential diagnosis, pathological speculation and prognosis prediction. However, no objective and wide-accepted criterion has been built up to now. The purpose of this study is to explore the optimal threshold that can be used for the detection and quantification of solid components in SSNs by using threshold segmentation method on computed tomography (CT) images.

**Methods:**

CT images of 102 SSNs were retrospectively analyzed. To establish a reference standard, the observers made judgments on whether the solid component existed in every SSN and did manual measurements of the volume of solid component with the help of software. Threshold segmentations of every nodule were then performed using different threshold settings and all of the measured volumes were assumed to be solid volumes, then solid-tototal volume ratios were calculated. The results were compared with the reference standards using the receiver operating characteristic curve and *Wilcoxon* test.

**Results:**

The application of thresholds as -250 HU or -300 HU resulted in high diagnostic value on the detection of solid component, with area under curve values as 0.982 and 0.977, respectively; the cut-off values of solid-to-total volume ratio were 1.10% and 6.14%, respectively; the median volumes of solid components were 202.7 mm^3^ (598.2 mm^3^), 247.1 mm^3^(696.0 mm^3^), which were not significantly different from the reference standard[199.5 mm^3^ (743.1 mm^3^)](*P*=0.125, 1, 0.061, 3).

**Conclusion:**

Threshold segmentation on chest CT images is valuable to detect and quantify the solid component on SSNs, the thresholds as -250 HU and -300 HU are recommended.

在胸部计算机断层扫描（computed tomography, CT）图像上肺内亚实性结节（subsolid nodule, SSN）是指纯磨玻璃结节和部分实性结节，病变完全呈磨玻璃密度的为纯磨玻璃结节，同时含实性和磨玻璃密度的为部分实性结节^[1, 2]^。现有研究表明，持续存在的SSN与肺腺癌密切相关^[3-5]^，在CT图像上显示出的SSN内实性成分的大小与肿瘤浸润成分的大小密切相关^[6, 7]^。但目前对SSN内实性成分做CT体积定量的研究报告尚少^[8, 9]^。本研究拟以观察者判定的SSN类型和CT半自动分割所得实性成分体积为参照标准，确定CT阈值分割法识别SSN实性成分并定量其体积的最佳阈值。

## 1 材料与方法

### 1.1 研究对象

回顾性分析2012年1月-2016年12月间在天津医科大学总医院医学影像科行胸部多层螺旋CT（multi-slice spiral CT, MSCT）检查发现的SSN病例。筛选出采用常规辐射剂量、重建层厚为1.25 mm的平扫图像数据，确保病变所在层面显示清晰、无明显呼吸运动伪影且病变轴位最大径在5 mm-30 mm。共获156例结节，其中51例结节因被较粗支气管/血管（管径2 mm以上）穿过、与较粗血管/胸膜相贴或在连续轴位图像上不能始终为一个整体而被剔除。最终入组来自94位患者（男性26位、女性68位）的102例结节，患者平均年龄（62±14）岁，结节轴位最大径（18±7）mm。

### 1.2 CT采集和重建方法

使用64层（Discovery HD CT、Light Speed VCT、Optima）或16层（Light Speed）MSCT（General Electric Company, GE），扫描范围自胸廓入口至肺底。患者一次吸气后屏气完成全肺扫描，螺旋扫描方式，120 kV或140 kV，200 mA-340 mA或噪声指数自动调制，螺距默认值1.375:1，机架旋转一周0.4 s-0.7 s，显示野（field of view, FOV）默认值360 mm，图像矩阵512×512，采用标准算法重建，获得1.25 mm层厚连续轴位无重叠图像。

### 1.3 CT图像观察及后处理测量

所有图像均在GE AW4.6工作站进行分析。结节分析在1.25 mm层厚肺窗图像上进行，窗宽1, 500 HU、窗位-700 HU。由两位医生（观察者1、观察者2）分别独立对结节内有无实性成分，即结节的类型（部分实性或纯磨玻璃）进行主观判断，结果不一致时采纳第三位医生（观察者3）的意见，由此确定出所有结节的类型并以此作为评估阈值分割法识别结节内实性成分效能的参照标准。判断病变为实性密度的标准为密度增高完全掩盖血管、间质结构；磨玻璃密度为雾样密度增高，但未掩盖其中血管、间质结构，纯磨玻璃结节可含有表现为实性密度的正常血管、间质等成分^[1, 2]^。被判定为部分实性的结节由观察者1和观察者2分别独立对其实性成分进行体积测量。观察者在轴位图像上确定目标结节，借助Auto Contour软件包在较靠近实性成分中心且密度较高的部位标记种籽点，软件自动勾画出实性成分的轮廓，手动逐层对其进行调整，满意后由计算机给出实性成分的体积。以两位观察者所得实性成分体积的平均值作为评估阈值分割法定量SSN实性成分体积的参照标准。观察者1对结节进行阈值分割（图 1），首先采用Auto Contour软件包对结节进行整体提取并记录结节的整体体积，然后使用3D-color-ROI工具计算所获结节内不同CT值区间的体素体积。共设9个CT值区间，下限值（阈值）分别设为-500 HU、-450 HU、-400 HU、-350 HU、-300 HU、-250 HU、-200 HU、-160 HU、-130 HU^[10]^，上限值均设为2, 000 HU。假定上述CT值区间的体素均为实性成分。计算阈值分割所获实性成分体积与结节整体体积的比率（%）。

**1:**
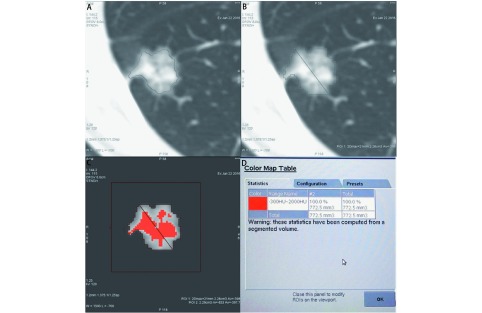
亚实性肺结节阈值分割示意图。A：标记结节后，软件自动勾画其轮廓；B：手动调整结节轮廓，满意后由计算机给出其体积；C、D：计算所分割结节内特定CT值区间的体素体积。 The threshold segmentation of a pulmonary subsolid nodule. A: After marking the nodule, the software can automatically draw a profile of the targeted nodule; B: Adjust the profile manually until satisfied, the computer can give the volume of the nodule; C, D: Quantify the volume of the segmented nodule using a certain threshold setting.

### 1.4 统计学分析

采用MedCalc 15.6统计软件对数据进行分析。符合正态分布的数据以均数±标准差（Mean±SD）表示，偏态分布数据以中位数（median, Md）[四分位间距（inter-quartile range, IQR）]表示。使用Cohen kappa评估主观判断结节类型的观察者间一致性。以观察者确定的结节类型为状态变量，以体积比率为检验变量，绘制不同CT值区间判断结节类型的受试者工作特征（receiver operating characteristic, ROC）曲线，得到曲线下面积（area under curve, AUC）。应用*DeLong*检验进行ROC曲线比较。通过Youden指数选出确认结节存在实性成分的体积比率界限值，体积比率≤界限值者判断为纯磨玻璃结节，> 界限值者判断为部分实性结节。应用组内相关系数（intraclass correlation coefficient, ICC）检验比较两观察者间实性成分半自动分割所得体积的一致性。对阈值分割所得体积与实性成分体积参照标准之间的差异性进行配对Wilcoxon检验。以*P* < 0.05作为有统计学差异的标准。

## 2 结果

### 2.1 观察者判定SSN的类型

102例SSN类型判断的观察者间一致性高，Cohen kappa值为0.87（表 1）。有6例结节观察者1和观察者2意见不一致，观察者3判断其中2例为部分实性结节，4例为纯磨玻璃结节。最终判定部分实性结节63例，纯磨玻璃结节39例。

**1:** 亚实性肺结节类型观察者判定（*n*=102） Observers' evaluation of SSNs (*n*=102)

		Observer1		Total
		Part solid	Pure ground glass	
Observer 2	Part solid	61	2	63
	Pure ground glass	4	35	39
	Total	65	37	102

### 2.2 阈值分割法判断SSN的类型

阈值分割法判断SSN类型的效能取决于所选的阈值。以观察者确定的结节类型作为参照标准，阈值从-500 HU到-130 HU对应的ROC曲线下面积呈先逐渐增大后逐渐减小的趋势，其中阈值为-250 HU时AUC最大（0.982），判断SSN类型的准确度最高（敏感度、特异度分别为100.0%和89.7%），对应最大Youden指数（0.897）的体积比率为1.10%；阈值为-300 HU时AUC次之（0.977），敏感度、特异度分别为90.5%和94.9%，对应最大Youden指数（0.854）的体积比率为6.14%。阈值-250 HU与-350 HU、-300 HU、-200 HU、-160 HU、-130 HU对应的ROC曲线间差异均不显著（*P*均 > 0.05），并且对应AUC值均大于0.9，即以上阈值在判断SSN类型时均具有较高的效能（表 2）。

**2:** 不同阈值判断亚实性结节类型的效能（*n*=102） Performance of subsolid nodules classification with different threshold settings (*n*=102)

Threshold	AUC	*P*	Cut-off value	Sens	Spec	Largest Youden index
-500 HU	0.922	0.003, 4	29.79%	85.7%	79.5%	0.652
-450 HU	0.945	0.014, 3	18.66%	88.9%	84.6%	0.735
-400 HU	0.962	0.046, 2	7.56%	98.4%	82.1%	0.805
-350 HU	0.971	0.098, 9	4.87%	98.4%	84.6%	0.830
-300 HU	0.977	0.239, 8	6.14%	90.5%	94.9%	0.854
-250 HU	0.982	Reference	1.10%	100.0%	89.7%	0.897
-200 HU	0.976	0.425, 8	0.79%	96.8%	92.3%	0.891
-160 HU	0.969	0.252, 6	1.71%	90.5%	97.4%	0.879
-130 HU	0.962	0.111, 7	0.12%	95.2%	92.3%	0.875
AUC: area under the curve; Sens: sensitivity; Spec: specificity.						

### 2.3 阈值分割法定量实性成分体积

63例部分实性结节中剔除实性成分内有空泡（*n*=12）、在连续轴位图像上实性成分不能始终为一个整体（*n*=4）、实性成分边界模糊（*n*=3）和实性成分轴位最长径不足3 mm（*n*=1）的结节^[11]^，最终入组43例部分实性结节，实性成分轴位最大径10 mm（10 mm）。两观察者实性成分半自动分割所得体积间的一致性好，ICC为0.97，实性成分体积分别为208.0 mm^3^（690.0 mm^3^）和206.0 mm^3^（820.0 mm^3^），平均值为199.5 mm^3^（743.1 mm^3^）。配对*Wilcoxon*检验显示阈值为-250 HU和-300 HU时所得实性成分体积与参照标准之间无显著差异（*P*=0.125, 1, 0.061, 3），而其他阈值所得实性成分体积均与参照标准间存在显著差异（*P*均 < 0.05）（表 3）。

**3:** 部分实性结节实性成分体积定量的阈值分割与参考标准比较(*n*=43) Comparison between threshold segmentations and the reference standard in volume quantification of solid components in part solid nodules (*n*=43)

Threshold	Md volume (mm^3^)	IQR (mm^3^)	*P*
-500 HU	652.3	1252.6	< 0.000, 1
-450 HU	496.7	1081.3	< 0.000, 1
-400 HU	374.2	912.7	< 0.000, 1
-350 HU	295.3	811.4	0.000, 1
-300 HU	247.1	696.0	0.061, 3
-250 HU	202.7	598.2	0.125, 1
-200 HU	164.4	518.5	< 0.000, 1
-160 HU	135.4	467.2	< 0.000, 1
-130 HU	115.6	422.1	< 0.000, 1
Reference standard	199.5	743.1	-
Md: median; IQR: inter-quartile range.			

## 3 讨论

SSN实性成分的识别与定量对SSN的鉴别诊断，病理预测和预后评估具有重要价值，因此越来越受到重视^[12-16]^，但目前缺乏公认且客观的标准。三维定量评估与二维定量评估^[6, 7, 10, 17-19]^相比能更充分地利用结节的信息且可实现结节内部成分的区分，同时重复性较好，但目前相关研究报告尚少^[8, 9]^。本研究对CT图像中整体半自动提取的SSN进行阈值分割，能够较准确、可靠地对其实性成分进行评估。

本研究证实，当阈值为-250 HU、体积比率 > 1.10%时判断SSN内存在实性成分的效能最高（AUC 0.982、敏感度100.0%、特异度89.7%）；当阈值为-300 HU、体积比率 > 6.14%时效能次之（AUC 0.977、敏感度90.5%、特异度94.9%）。这与Yanagawa等^[8, 20]^得出的部分实性结节内所含 > -291 HU的体素至少应达2%的结论一致。纯磨玻璃结节内表现为实性密度的正常血管、间质等成分会被误判为实性成分病变，体积比率界限值的存在即反映了这种情况，此界限值随阈值的增高而呈减小趋势。

本研究以部分实性结节实性成分两观察者半自动分割所得体积的平均值为参照标准，得出阈值分割中可选择-250 HU和-300 HU为阈值来定量结节内实性成分的体积。本结论与Yanagawa等^[20]^（ > -291 HU）、Scholten等^[10]^（-300 HU）和Cohen等^[6]^（-350 HU和-400 HU）的结论也相似。

本研究有如下局限性。首先，缺乏与病理的对照。其次，本研究中用于半自动分割的Auto Contour软件包难以准确分析连续轴位图像上不能始终为一个整体的结节及其实性成分。

综上所述，CT阈值分割能够可靠地识别SSN内的实性成分并对其体积进行定量评估；阈值可设为-250 HU或-300 HU，对应结节分类体积比率界限值分别为1.10%、6.14%。
